# Commonality in dysregulated expression of gene sets in cortical brains of individuals with autism, schizophrenia, and bipolar disorder

**DOI:** 10.1038/s41398-019-0488-4

**Published:** 2019-05-24

**Authors:** Jinting Guan, James J. Cai, Guoli Ji, Pak Chung Sham

**Affiliations:** 1 Department of Automation, Xiamen University, Xiamen, China; 2 National Institute for Data Science in Health and Medicine, Xiamen University, Xiamen, China; 3 Department of Veterinary Integrative Biosciences, Texas A&M University, College Station, TX USA; 4 Interdisciplinary Program in Genetics, Texas A&M University, College Station, TX USA; 5 Innovation Center for Cell Signaling Network, Xiamen University, Xiamen, China; 6 Department of Psychiatry, The University of Hong Kong, Hong Kong, China; 7 Centre for Genomics Science, Li Ka Shing Faculty of Medicine, The University of Hong Kong, Hong Kong, China

**Keywords:** Autism spectrum disorders, Schizophrenia, Bipolar disorder

## Abstract

Individuals affected with different neuropsychiatric disorders such as autism (AUT), schizophrenia (SCZ) and bipolar disorder (BPD), may share similar clinical manifestations, suggesting shared genetic influences and common biological mechanisms underlying these disorders. Using brain transcriptome data gathered from postmortem donors affected with AUT, SCZ and BPD, it is now possible to identify shared dysregulated gene sets, i.e., those abnormally expressed in brains of neuropsychiatric patients, compared to non-psychiatric controls. Here, we apply a novel aberrant gene expression analysis method, coupled with consensus co-expression network analysis, to identify gene sets with shared dysregulated expression in cortical brains of individuals affected with AUT, SCZ and BPD. We identify eight gene sets with dysregulated expression shared by AUT, SCZ and BPD, 23 by AUT and SCZ, four by AUT and BPD, and two by SCZ and BPD. The identified genes are enriched with functions relevant to amino acid transport, synapse, neurotransmitter release, oxidative stress, nitric oxide synthase biosynthesis, immune response, protein folding, lysophosphatidic acid-mediated signaling and glycolysis. Our method has been proven to be effective in discovering and revealing multigene sets with dysregulated expression shared by different neuropsychiatric disorders. Our findings provide new insights into the common molecular mechanisms underlying the pathogenesis and progression of AUT, SCZ and BPD, contributing to the study of etiological overlap between these neuropsychiatric disorders.

## Introduction

Autism (AUT), schizophrenia (SCZ) and bipolar disorder (BPD) are three major neuropsychiatric disorders. AUT patients present with impairments in social interaction and communication, and repetitive and restricted behaviors. SCZ is characterized by delusions, hallucinations, disordered thoughts and blunted affect. The symptoms of BPD include recurrent mania and depression, frequently with delusions. Patients with these severe neuropsychiatric disorders share similar behavioral, social, cognitive, and perceptual impairments. Up to 30% of individuals diagnosed with AUT during childhood will develop SCZ in adulthood^1^. The presence of SCZ or BPD in first-degree relatives is a consistent and significant risk factor for AUT^2^. Similarity in clinic symptoms, as well as shared genetic influences, between AUT, SCZ and BPD, have been the focus of several recent studies^3–7^.

Genetic studies have identified genetic variants that contribute to the risk of developing AUT^8–14^, SCZ^15–18^ and BPD^19–22^. However, for any of these disorders, it remains elusive how these reported variants lead to the pathogenesis of disorder. One possibility is that these variants cause gene expression alternations in brain (during a certain stage or across several stages of brain development) and eventually lead to the neuropsychiatric disorders. Measuring and analyzing gene expression information in postmortem brains is thus valuable for understanding the pathogenesis of neuropsychiatric disorders. The availability of samples from brains of diseased cases and healthy controls makes it possible to measure the gene expression from this primarily affected organ for understanding AUT^23,24^, SCZ^25,26^ and BPD^27,28^. To study the effect of genetic correlation in these disorders, some researchers focused on studying the overlap of gene expression alterations between diseases^29–32^. For example, a transcriptome analysis of anterior cingulate cortex samples from SCZ, BPD and controls was conducted by Zhao et al.^31^. They showed the gene expression of SCZ and BPD are correlated, and dysregulation of cytoskeleton remodeling and lysosomal function underlies the common causes of SCZ and BPD. In a more recent study, Ellis et al. integrated and analyzed the transcriptomic RNA-sequencing data of cortex samples of AUT, SCZ, BPD and controls combined from two previous studies^24,31^. Ellis et al. used univariate analysis method, i.e., single gene-based differential expression analysis, to first identify differentially expressed genes associated with each disease, and then obtained a list of differentially expressed genes shared by those disorders. They reported the transcriptomes of AUT and SCZ are correlated, and altered neurotransmission and synapse regulation are shared between these two disorders^32^.

To reveal shared dysregulated gene sets between AUT, SCZ and BPD, herein we re-analyze the gene expression data of cortical brain tissue samples from AUT, SCZ, BPD and healthy controls^32^ but applying multivariate analysis methods. We construct gene co-expression networks to find consensus modules shared across disorders. To consider the gene expression dispersion, we apply aberrant gene expression analysis to identify shared gene sets tend to be aberrantly expressed across diseases. By analyzing the shared dysregulated gene sets, we evaluate the extent of similarity between gene expression of AUT, SCZ and BPD and gain better understanding the downstream impact of genetic overlap in these neuropsychiatric disorders.

## Materials and methods

### Gene expression data

The gene expression data of 104 cortical brain tissue samples (47 AUT and 57 controls), which was normalized using conditional quantile normalization (CQN)^33^ to remove technical variability, was obtained from the study of Ellis et al.^32^. We denoted it as data I. These 104 samples were from three brain regions, of which 62, 14 and 28 were from cerebral cortex (BA 19), anterior prefrontal cortex (BA 10), and a part of the frontal cortex (BA 44) respectively, involving 40 healthy and 32 autistic individuals. The transcriptomes of the 104 samples were originally sequenced by Gupta et al.^24^. The CQN normalized gene expression data of 82 anterior cingulate cortex (BA24) samples (involving 31 SCZ patients, 25 BPD patients, and 26 controls) was also obtained from the study of Ellis et al.^32^, which we denoted as data II. These samples were originally from Stanley Medical Research Institute (SMRI) and the whole transcriptome sequencing was performed by Zhao et al.^31^. For these two datasets (data I and data II), the sequencing reads were subjected to a common pre-processing pipeline for obtaining the gene expression data^32^.

In this study, we first combined data I and data II, and processed the combined data using the algorithm of ComBat^34^ for removing the effect caused by different data sources and regressing out the covariates (age, gender and brain region). Next, we applied the algorithm of probabilistic estimation of expression residuals (PEER)^35^ to discover up to 20 possible hidden determinants of expression variation and then regressed out the hidden factors that were uncorrelated with disease status. The lowly expressed genes with expression median < 2 were excluded. The final data matrix contains the expression level of 8485 protein-coding genes in 186 samples (47 AUT, 31 SCZ, 25 BPD and 83 controls).

### Curated gene sets

The gene sets (*n* = 17,786) used in the Gene Set Enrichment Analysis (GSEA) were obtained from the molecular signatures database (MSigDB v6.1)^36^. The GSEA gene sets include 50 hallmark gene sets, 326 positional gene sets, 4738 curated gene sets, 836 motif gene sets, 858 computational gene sets, 5917 GO gene sets, 189 oncogenic signatures and 4872 immunologic signatures. A total of 1007 AUT-candidate genes were downloaded from gene scoring module in Simons Foundation Autism Research Initiative (SFARI), which includes 68, 25, 59, 176, 406, 157, and 21 genes from categories S (syndromic), 1 (high confidence), 2 (strong candidate), 3 (suggestive evidence), 4 (minimal evidence), 5 (hypothesized but untested) and 6 (evidence does not support a role). We downloaded 2752 genes associated with SCZ from SZDB^37^, database for schizophrenia genetic research, and these genes were identified by different kinds of studies including convergent functional genomics, CNV, differentially expression, GWAS, genetic linkage and association studies, *Sherlock* integrative analysis and *Pascal* gene-based test. We also downloaded 599 BPD-associated genes from BDgene database^38^, and each gene is positively supported by at least one kind of studies.

### Aberrant gene expression analysis

The aberrant gene expression analysis^39^ is a multivariate method, which adopts Mahalanobis distance (MD)^40^ to quantify the dissimilarity in multigene expression patterns between diseased samples and control group. Here we firstly applied aberrant gene expression analysis to identify gene sets that may be expressed aberrantly in each of the three disorders (AUT, SCZ and BPD). Specifically, based on the final data matrix including 186 samples (47 AUT, 31 SCZ, 25 BPD and 83 controls), we first calculated, for each disorder and each given gene set, the MD from each diseased sample *i* to the robust multivariate centroid of control group (including 83 controls), denoted as MD_*i*_. MD measures the number of standard deviations from case sample to the robust mean of controls. To reduce the influence of possible outliers in control group, we adopted the algorithm of Minimum Covariance Determinant (MCD)^41^ to obtain the robust location estimator (expression mean) and scattering estimator (covariance matrix) of the controls. The MCD algorithm subsamples *h* observations (set *h* = 0.8*n*, where *n* is the number of controls) whose covariance matrix had the smallest covariance determinant, and the MCD robust estimates of location and scattering were imputed from these *h* controls. Then *MD*_*i*_ was calculated as:

$$MD_i = \sqrt {(x_{i \cdot } - y_c)^T\psi _c^{ - 1}(x_{i \cdot } - y_c)}$$

where *x*_*i∙*_ is the vector of gene expression levels for diseased sample *i*, *y*_*c*_ is the vector of expression means of genes across *h* control samples, and *ψ*_*c*_ is the covariance matrix estimated from the *h* controls.

Next, the sum of squared *MD*_*i*_, denoted as *SSMD*:

$$SSMD = \mathop {\sum}\nolimits_{i = 1}^m {MD_i^2}$$

was calculated to measure the overall dispersion of *m* cases to the robust centroid of control group. To assess the significance of *SSMD* of a given gene set, we performed permutation tests using *N* randomly reconstructed gene sets with the same size. As *SSMD* measures the overall dispersion of cases relative to the control group, even if the robust centroid of controls may change when we performed permutation tests, but it would not affect the calculation of a relative measure, i.e., *SSMD*. The *P*-value of permutations, *P*_perm_, was determined by *M*/*N*, where *M* is the number of random gene sets whose *SSMD* values are greater than that of the given gene set, *N* is the total number of random gene sets. The correction for multiple testing was performed by controlling the false discovery rate (FDR) with the Benjamini–Hochberg method^42^.

To assess the relative contribution of each gene in a significant gene set to the total *SSMD*, we calculated the difference between the total *SSMD* value and the *SSMD* value calculated after the gene was excluded from the gene set, which we denoted as Δ*SSMD*. The Δ*SSMD* of gene *j* was calculated as:

$${\mathrm{\Delta }}SSMD_j = SSMD - SSMD_{exclude\ gene \,j}$$

Then we sorted the genes by their Δ*SSMD* values for a significant gene set.

### Clustering of aberrantly expressed gene sets

For any pair of significant aberrantly expressed gene sets *a* and *b*, we adopted Jaccard distance^43^ to measure their dissimilarity, which was calculated as:

$$1 - J(a,b) = 1 - \frac{{\left| {a \cap b} \right|}}{{\left| {a \cup b} \right|}}$$

where *J*(*a*, *b*) is Jaccard similarity coefficient and is defined as the number of genes in the intersection of *a* and *b* divided by the number of genes in the union of *a* and *b*. Based on the Jaccard distance, we generated a hierarchical cluster tree to group the gene sets whose distances are less than 0.3.

### Consensus gene co-expression network analysis

In addition to aberrant gene expression analysis, we also performed gene co-expression network analysis. We first split the final data matrix into three sub-datasets, denoted as datasets 1, 2 and 3, each containing the data for a disorder and their respective controls. Then weighted gene co-expression network analysis (WGCNA)^44^ was applied to find consensus modules between datasets 1, 2 and 3. The signed consensus modules were built using the function of *blockwiseConsensusModules* in WGCNA package^45^. Modules were defined using biweight midcorrelation (bicor) which is more robust to outliers compared to Pearson correlation^46^, along with the soft-threshold power of 5 for all datasets achieving approximate scale-free topology (*R*^2^ > 0.8), minimum module size of 20 and FALSE pamStage. The module eigengene (the first principal component) was used to represent the expression level of each consensus module and was associated with the traits (age, gender, and disease status) to compute the correlation coefficients and *P*-values. The *P*-values were then corrected by controlling the FDR with the Benjamini–Hochberg method^42^. Gene ontology analysis was performed using the database for annotation, visualization and integrated discovery (DAVID)^47,48^. Gene lists of modules were uploaded, and the GO terms whose Bonferroni-adjusted *P*-value < 0.05 were reported as significant in the ‘Functional Annotation Chart’ generated using the minimum number of genes involved in the term of 10.

## Results

### Analysis workflow

**Fig. 1:**
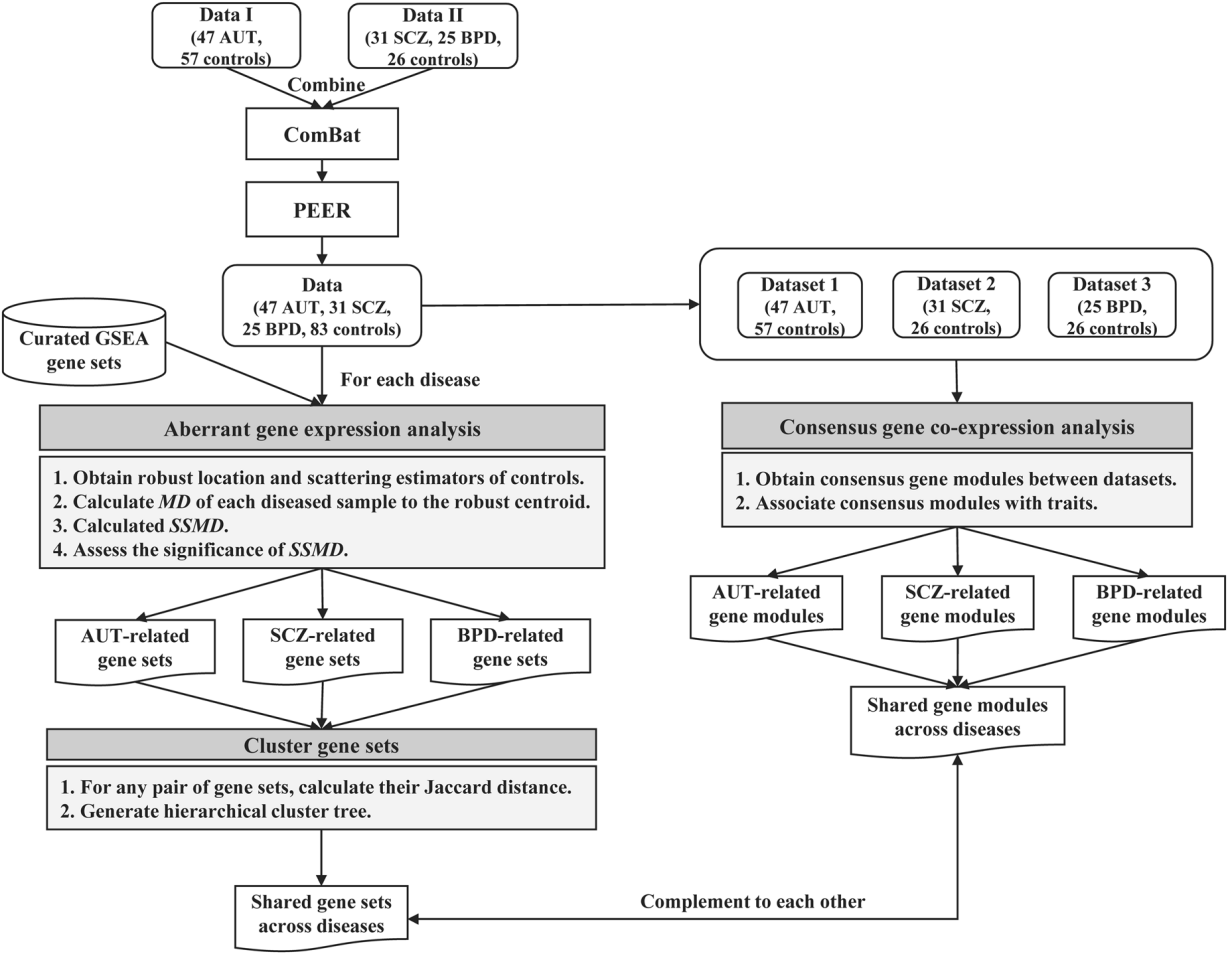
The analysis workflow used in parallel, including aberrant gene expression analysis and consensus gene co-expression analysis

Figure 1 describes the analysis workflow. The analyses of this study were conducted with the gene expression datasets, normalized using conditional quantile normalization (CQN)^33^ to remove technical variability, from the study of Ellis et al.^32^, including (1) the gene expression data of 104 cortical brain tissue samples (47 AUT and 57 controls), here denoted as data I, and (2) the gene expression data of 82 anterior cingulate cortex samples (31 SCZ, 25 BPD, and 26 controls), denoted as data II. Firstly, we combined data I and data II and processed the combined data with the algorithm of ComBat^34^ to remove batch effect. Then the data was subjected to the algorithm of probabilistic estimation of expression residuals (PEER)^35^ to regress out up to 20 possible hidden factors that were uncorrelated with disease status (Materials and Methods). The final data matrix contains the expression level of 8,485 protein-coding genes in 186 samples (47 AUT, 31 SCZ, 25 BPD and 83 controls) (Supplementary Data 1).

Next, we applied the aberrant gene expression analysis^39^ to identify significant gene sets for each disorder and then obtained shared ones across disorders by generating a hierarchical cluster tree. In addition, we used weighted gene co-expression network analysis (WGCNA)^44^ to find consensus modules shared across disorders. We then compared the results from these two kinds of analysis methods, aberrant gene expression analysis and WGCNA. By analyzing the identified shared sets of dysregulated genes, we assessed the commonality between AUT, SCZ, and BPD.

### Shared aberrantly expressed gene sets across disorders

**Fig. 2:**
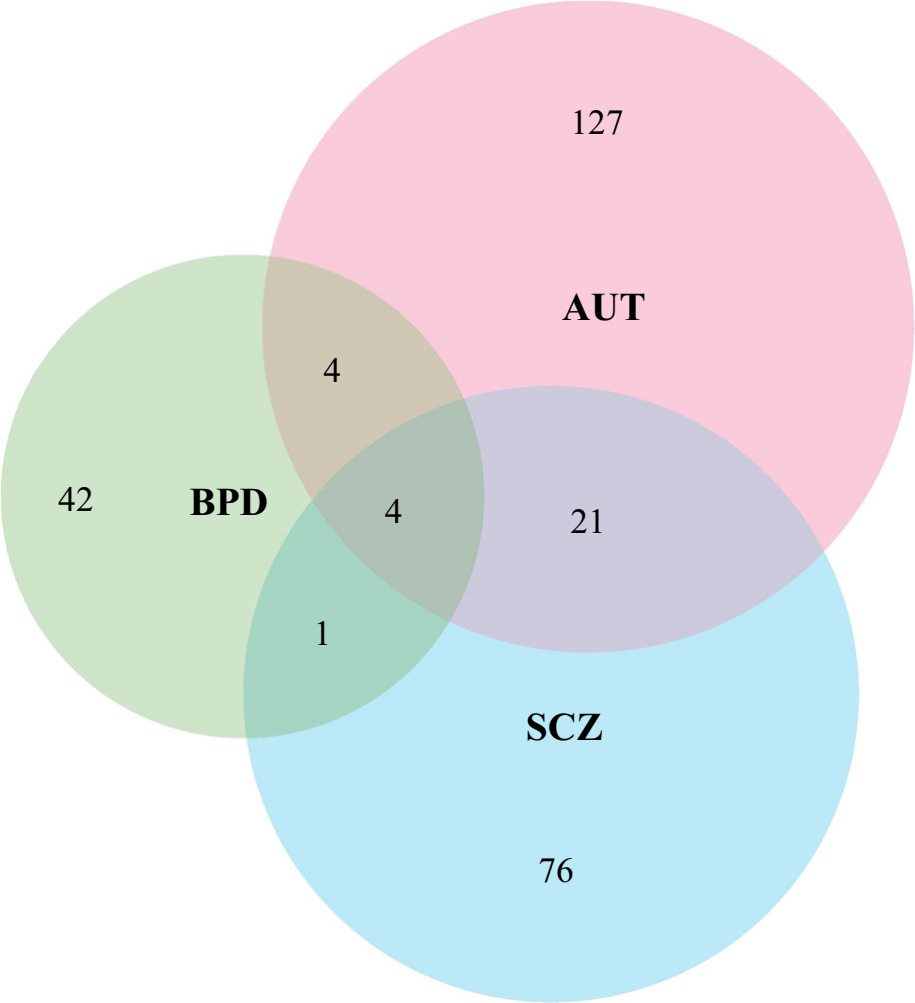
The Venn diagram shows the number of identified aberrantly expressed gene sets associated with AUT, SCZ, and BPD

Dysregulated gene expression is characterized by increased level of expression dispersion between individuals^23,39^. To assess the gene expression dispersion in diseased individuals, we used aberrant gene expression analysis method^39^ to identify gene sets tend to be aberrantly expressed in AUT, SCZ and BPD separately based on the final data matrix including 47 AUT, 31 SCZ, 25 BPD samples and 83 controls (Materials and Methods). From 17,786 GSEA (Gene Set Enrichment Analysis) gene sets^36^, 156, 102, 51 gene sets were identified to be associated with AUT, SCZ and BPD respectively (*P*-value ≤ 0.01) (Fig. 2). To consider the effect of different case sample sizes for different diseases, we re-run the aberrant gene expression analysis by using a same diseased sample size (i.e., the minimum sample size among AUT, SCZ and BPD cases, which is equal to 25). In specific, we randomly picked 25 case samples from all AUT cases, and used their gene expression to perform aberrant gene expression analysis. This process was repeated for 100 times, and we found that the average number of identified gene sets associated with AUT is 137. For SCZ, we also performed aberrant gene expression analysis for 100 times by using the gene expression of 25 randomly selected SCZ cases in each time. We found that the average number of identified gene sets associated with SCZ is 97. It can be seen that after we controlled the effect of different case sample sizes, the differences in the number of identified gene sets associated with different disorders still exist, especially between AUT and BPD, SCZ and BPD. It may because for our analyzed gene expression data AUT cases tend to show disruption in more gene sets or more biological functions relative to SCZ, and especially BPD.

Then we clustered the significant gene sets (156, 102 and 51 gene sets associated with AUT, SCZ and BPD respectively) to obtain shared ones by more than one disorders (Materials and Methods). As a result, there are 21 gene sets shared by AUT and SCZ, four shared by AUT and BPD, one shared by SCZ and BPD, four shared by all three disorders (Fig. 2). The shared GSEA gene sets are listed in Table 1. When the cutoff of *P*-value was set to 0.05, 119 gene sets are shared by AUT and SCZ, 44 shared by AUT and BPD, 25 shared by SCZ and BPD, 31 shared by all three disorders (Supplementary Table 1). To assess the overlap between genes in identified gene sets and disease-associated genes, we downloaded 1,007 AUT-associated genes from Simons Foundation Autism Research Initiative (SFARI), 2752 SCZ candidate genes from SZDB^37^ and 599 BPD candidate genes from BDgene^38^ (Materials and Methods). Many dysregulated gene sets are enriched with disease-associated genes (Table 1). To measure the relative contribution of each gene in a significant gene set, we calculated Δ*SSMD* value of each gene (Materials and Methods) and sorted the genes by their Δ*SSMD* values. For each disease, the genes with top three Δ*SSMD* values are listed for each significant gene set, and the ones overlapping with disease-associated genes are bold (Table 1**)**.

**Table 1:** The identified shared GSEA gene sets from aberrant gene expression analysis

Gene set name	Number of Genes	Shared by	Number of associated genes (*P*-value)			Genes with top three Δ*SSMD* values (100% × Δ*SSMD*/SSMD)			Reference
			AUT	SCZ	BPD	AUT	SCZ	BPD	
**GO_AMINO_ACID_TRANSMEMBRANE_TRANSPORTER_ACTIVITY**	34/79	AUT, SCZ	9 (0.0030)	16 (0.0020)	3 (0.0389)	*SLC6A15* (15.04%), *SLC43A2* (9.31%), *SLC25A22* (7.40%)	*SLC6A6* (13.91%), ***SLC38A5*** (13.84%), *SLC36A1* (12.09%)	*SLC25A12* (10.90%), *SLC6A1* (10.80%), *SLC7A8* (9.21%)	49–52
**GO_REGULATION_OF_OXIDATIVE_STRESS_INDUCED_INTRINSIC_APOPTOTIC_SIGNALING_PATHWAY**	23/29	AUT, SCZ	2 (0.2459)	6 (0.039)	3 (0.0103)	*HSPH1* (25.36%), *UBQLN1* (17.83%), *FBXW7* (13.65%)	*SFPQ* (13.58%), ***HSPB1*** (13.45%), *SOD2* (13.30%)	*P4HB* (17.10%), *HSPH1* (15.62%), *UBQLN1* (11.61%)	70–72
**GO_NEGATIVE_REGULATION_OF_OXIDATIVE_STRESS_INDUCED_INTRINSIC_APOPTOTIC_SIGNALING_PATHWAY**	16/21	AUT, SCZ	1 (0.3399)	6 (0.0045)	3 (0.0026)	*HSPH1* (19.12%), *HSPB1* (19.08%), *SOD2* (18.75%)	***HSPH1*** (21.68%), ***HSPB1*** (17.89%), *SOD2* (11.82%)	*HSPH1* (23.67%), *HSPB1* (13.80%), ***AKT1*** (11.23%)	70–72
**REACTOME_GLYCOLYSIS**	19/29	AUT, SCZ	2 (0.1664)	5 (0.2923)	1 (0.1618)	***PPP2R5D*** (15.30%), *PGAM1* (14.51%), *PFKFB2* (13.60%)	*PPP2R5D* (23.04), *PPP2CB* (16.19%), *GPI* (15.19%)	*PPP2CB* (18.48%), *PFKFB2* (16.64%), *PFKFB3* (14.21%)	94–96
**GO_NEUTRAL_AMINO_ACID_TRANSPORT**	19/34	AUT, SCZ	2 (0.1664)	7 (0.0032)	2 (0.0337)	*SLC38A5* (20.32%), *SLC32A1* (17.56%), *SLC6A15* (16.75%)	***SLC38A5*** (24.24), ***SLC32A1*** (23.77%), *SLC7A8* (21.49%)	*SLC43A2* (17.03%), *SLC6A15* (12.56%), *SLC7A8* (10.63%)	49–52
**REACTOME_NEUROTRANSMITTER_RELEASE_CYCLE**	27/34	AUT, SCZ	13 (1.57E-8)	13 (0.0002)	6 (0.0004)	*GLS* (16.82%), *RAB3A* (13.80%), *SLC1A6* (13.16%)	*SLC6A12* (9.05%), ***HSPA8*** (8.48), *ALDH5A1* (7.46%)	*GAD2* (18.88%), *ALDH5A1* (14.37%), ***GAD1*** (14.28%)	57
REACTOME_JNK_C_JUN_KINASES_PHOSPHORYLATION_AND_ACTIVATION_MEDIATED_BY_ACTIVATED_HUMAN_TAK1	10/16	AUT, SCZ	0 (0.5414)	1 (0.4367)	0 (0.3218)	*MAPK9* (25.17%), *TAB1* (18.19%), *MAP2K4* (17.56%)	*IRAK1* (29.17%), ***MAPK9*** (20.91%), *TAB1* (19.21%)	*MAPK9* (21.92%), *IRAK1* (18.64%), *TAB1* (17.60%)	
BIOCARTA_NDKDYNAMIN_PATHWAY	16/21	AUT, SCZ	2 (0.1131)	3 (0.4150)	1 (0.1222)	*EPN1* (15.65%), *AMPH* (12.16%), ***BIN1*** (11.53%)	*EPN1* (34.05%), *CALM3* (25.69%), *PPP3CA* (17.24%)	*EPN1* (25.64%), *EPS15* (14.80%), *PICALM* (13.19%)	
**GO_NEGATIVE_REGULATION_OF_CATECHOLAMINE_SECRETION**	5/16	AUT, SCZ	6 (0.0037)	6 (0.0019)	3 (0.1764)	***ABAT*** (35.25%), *ADRA2A* (27.67%), *CHGA* (25.70%)	*ABAT* (35.40%), ***CNR1*** (35.02%), *ADRA2A* (28.29%)	*CNR1* (33.40%), *ABAT* (33.06%), *ADRA2A* (30.65%)	63–65
**GO_POSITIVE_REGULATION_OF_NITRIC_OXIDE_SYNTHASE_BIOSYNTHETIC_PROCESS**	2/14	AUT, SCZ	0 (0.1443)	5 (0.0210)	4 (0.0014)	*MAPK9* (69.64%), *NAMPT* (43.63%),	***MAPK9*** (79.72%), *NAMPT* (25.30%)	*MAPK9* (85.87%), ***NAMPT*** (14.47%)	71,74–77
**GO_REGULATION_OF_NITRIC_OXIDE_SYNTHASE_BIOSYNTHETIC_PROCESS**	3/19	AUT, SCZ	0 (0.2085)	6 (0.0568)	4 (0.0042)	*NAMPT* (36.75%), *MAPK9* (28.79%), *GSTP1* (19.81%)	***MAPK9*** (42.53%), *NAMPT* (25.36%), *GSTP1* (20.25%)	*MAPK9* (44.01%), *GSTP1* (28.57%), ***NAMPT*** (11.78%)	71,74–77
KEGG_PRION_DISEASES	17/35	AUT, SCZ	4 (0.0339)	8 (0.0874)	4 (0.0250)	*ELK1* (14.45%), *HSPA5* (13.45%), *PRNP* (13.04%)	***EGR1*** (18.08%), *MAPK1* (13.14%), *ELK1* (12.18%)	*HSPA5* (19.20%), *ELK1* (15.93%), *EGR1* (15.65%)	
**GO_L_AMINO_ACID_TRANSMEMBRANE_TRANSPORTER_ACTIVITY**	25/54	AUT, SCZ	6 (0.0353)	13 (0.0016)	3 (0.0138)	*SLC38A5* (18.11%), *SLC6A15* (16.75%), *SERINC3* (13.94%)	*SERINC3* (18.89%), ***SLC38A5*** (17.91%), *SLC36A1* (15.69%)	*SLC43A2* (17.06%), *SLC38A5* (16.14%), *SLC36A4* (13.91%)	49–52
**GO_L_AMINO_ACID_TRANSPORT**	29/58	AUT, SCZ	8 (0.0047)	14 (0.0004)	3 (0.0231)	*SERINC3* (16.86%), *SLC7A8* (16.65%), *SLC36A4* (13.75%)	*SLC7A8* (19.52%), ***SLC38A5*** (18.49%), ***SLC32A1*** (13.92%)	*SERINC3* (17.51%), *SLC36A4* (14.88%), *SLC7A8* (14.30%)	49–52
**REACTOME_AMINO_ACID_TRANSPORT_ACROSS_THE_PLASMA_MEMBRANE**	14/31	AUT, SCZ	3 (0.2829)	6 (0.0408)	0 (0.4194)	*SLC38A5* (20.77%), ***SLC7A5*** (19.53%), *SLC3A2* (17.81%)	***SLC38A5*** (22.59%), *SLC38A2* (14.81%), *SLC7A5* (14.00%)	*SLC43A2* (13.49%), *SLC6A15* (12.37%), *SLC38A5* (10.94%)	49–52
**GO_GLUTAMATE_SECRETION**	27/28	AUT, SCZ	9 (1.36E-5)	9 (0.0032)	7 (4.76E-6)	*SLC17A7* (12.71%), ***SLC1A1*** (12.19%), *PPFIA4* (11.87%)	***RIMS1*** (12.46%), *SLC38A2* (10.67%), *GIPC1* (8.17%)	*UNC13B* (20.75%), *CPLX1* (14.43%), *SLC17A7* (14.37%)	59–62
**ST_PAC1_RECEPTOR_PATHWAY**	5/7	AUT, SCZ	0 (0.3227)	0 (0.5428)	0 (0.1764)	*ASAH1* (43.59%), *DAG1* (40.54%), *ITPKA* (33.57%)	*DAG1* (36.17%), *ITPKB* (35.09%), *ITPKA* (34.83%)	*DAG1* (30.20%), *ASAH1* (29.48%), *ITPKB* (28.75%)	101–104
**GO_INHIBITORY_SYNAPSE**	8/12	AUT, SCZ	2 (0.0176)	2 (0.3265)	1 (0.2670)	*IQSEC3* (33.44%), *SLC32A1* (31.26%), *GAD2* (28.51%)	*BSN* (20.42%), *NPTN* (18.29%), *NLGN3* (7.72%)	*GAD2* (24.48%), *IQSEC3* (22.83%), *SLC32A1* (15.91%)	66–68
**GO_SYNAPTIC_VESICLE_RECYCLING**	20/23	AUT, SCZ	2 (0.1856)	3 (0.5699)	1 (0.1755)	*SH3GL2* (18.31%), *SYT5* (12.87%), ***SYNJ1*** (11.79%)	*PACSIN1* (15.57%), *SYT2* (12.31%), *CANX* (10.92%)	*RAB5A* (14.77%), ***GRN*** (14.25%), *CANX* (13.74%)	53–56
**GO_SYNAPTIC_VESICLE_ENDOCYTOSIS**	17/17	AUT, SCZ	2 (0.1301)	2 (0.4558)	1 (0.1351)	*SH3GL2* (14.40%), *SYT1* (11.25%), *SYT2* (10.06%)	*SYT2* (19.04%), *CANX* (17.58%), *SYT12* (13.76%)	***GRN*** (21.94%), *CANX* (14.44%), *SCRIB* (13.51%)	53–56
GO_RESPONSE_TO_COLD	22/43	AUT, SCZ	5 (0.2254)	7 (0.6371)	5 (0.0493)	*VGF* (13.57%), *PPARGC1A* (11.54%), *HSP90AA1* (10.43%)	*VGF* (11.16%), *CIRBP* (8.30%), *PCSK1N* (7.21%)	*EIF2AK4* (14.25%), *ATP2B1* (9.27%), *FOXO1* (9.26%)	
**GO_CAMP_BIOSYNTHETIC_PROCESS**	7/17	AUT, BPD	3 (0.0117)	4 (0.2690)	4 (6.59E-5)	***ADCY5*** (26.57%), *ADCY9* (20.36%), ***ADCY3*** (19.20%)	*ADCY5* (30.68%), ***ADCY8*** (26.01%), *ADCY9* (20.13%)	*ADCY5* (43.90%), ***ADCY8*** (18.44%), ***ADCY2*** (16.09%)	97–99
**GO_CELL_ACTIVATION_INVOLVED_IN_IMMUNE_RESPONSE**	41/139	AUT, BPD	10 (0.1903)	22 (0.0111)	4 (0.2040)	*PLCL2* (8.35%), *RNF168* (7.74%), *S100A13* (7.50%)	*LGALS1* (21.57%), *PLCL2* (15.12%), *GBF1* (14.01%)	*TICAM1* (10.42%), *HSPD1* (9.08%), *PRKCE* (6.14%)	78–81
**GO_LYMPHOCYTE_ACTIVATION_INVOLVED_IN_IMMUNE_RESPONSE**	29/98	AUT, BPD	6 (0.1687)	17 (0.0179)	4 (0.0965)	*HSPD1* (16.48%), *PTK2B* (16.08%), *NOTCH2* (16.00%)	***HSPD1*** (17.79%), *EIF2AK4* (14.57%), *PTK2B* (9.98%)	*EIF2AK4* (21.75%), *PSEN1* (14.88%), *HSPD1* (14.61%)	78–81
GO_LYMPHOCYTE_CHEMOTAXIS	3/38	AUT, BPD	2 (0.2085)	2 (0.3747)	1 (0.1099)	*GAS6* (50.33%), *CX3CL1* (41.10%), *PIK3CD* (36.18%)	*GAS6* (44.40%), *CX3CL1* (38.61%), *PIK3CD* (21.99%)	*GAS6* (57.14%), *CX3CL1* (46.90%), *PIK3CD* (39.55%)	
GO_POSITIVE_REGULATION_OF_WOUND_HEALING	16/48	SCZ, BPD	5 (0.1131)	9 (0.4150)	4 (0.1222)	*PRKCE* (17.32%), *USF1* (16.93%), *ARFGEF1* (11.96%)	*PRKCE* (38.85%), *ARFGEF1* (22.08%), *EPB49* (21.36%)	*USF1* (15.18%), *PRKCE* (13.66%), *MYLK* (9.65%)	
**GO_DE_NOVO_POSTTRANSLATIONAL_PROTEIN_FOLDING**	12/14	AUT, SCZ, BPD	0 (0.6076)	6 (0.0005)	0 (0.3725)	*HSPH1* (28.95%), *DNAJB1* (16.38%), *HSPE1* (16.11%)	***DNAJB1*** (21.82%), ***HSPE1*** (17.90%), ***HSPH1*** (16.17%)	*HSPH1* (21.43), *HSPE1* (18.28%), *DNAJB1* (14.56%)	88,90,91
**GO_DE_NOVO_PROTEIN_FOLDING**	17/19	AUT, SCZ, BPD	0 (0.7344)	7 (0.0014)	0 (0.4834)	*CHCHD4* (11.84%), *DNAJB1* (10.57%), *HSPH1* (10.30%)	***DNAJB1*** (22.56%), ***HSPD1*** (14.58%), ***CHCHD4*** (11.04%)	*DNAJB1* (20.22%), *HSPD1* (10.96%), *CHCHD4* (10.21%)	88,90,91
GO_PROTEIN_TARGETING_TO_PLASMA_MEMBRANE	19/23	AUT, SCZ, BPD	4 (0.0112)	4 (0.5335)	1 (0.1618)	*INPP5K* (17.73%), *GAS6* (15.78%), *BSG* (15.72%),	*INPP5K* (16.30%), *MYADM* (14.08%), *EHD3* (12.05%)	*GAS6* (16.52%), *INPP5K* (14.40%), *PALM* (13.35%)	
**PID_LPA4_PATHWAY**	12/15	AUT, SCZ, BPD	2 (0.0554)	2 (0.2465)	4 (4.92E-5)	*ADCY8* (14.99%), *PRKCE* (12.68%), *RPS6KA5* (11.95%)	*PRKCE* (24.85%), ***RPS6KA5*** (22.22%), ***ADCY8*** (22.18%)	*ADCY5* (18.87%), ***ADCY8*** (18.13%), *PRKCE* (16.52%)	92,93

The numbers of included genes in the analyzed gene expression data and the number of total genes in the gene set are listed. The mentioned gene sets are bold. In each gene set, the number of genes which are overlapped with AUT, SCZ and BPD-associated genes is shown with hypergeometric test *P*-value. For each disease, the genes with top three Δ*SSMD* values are listed for each gene set, and the ones overlapping with disease-associated genes are bold

#### Shared gene sets involved in amino acid transport

In Table 1, five gene sets, associated with AUT and SCZ, are related to amino acid transport including *GO_AMINO_ACID_TRANSMEMBRANE_TRANSPORTER_ACTIVITY*, *GO_NEUTRAL_AMINO_ACID_TRANSPORT*, *GO_L_AMINO_ACID_TRANSMEMBRANE_TRANSPORTER_ACTIVITY*, *GO_L_AMINO_ACID_TRANSPORT* and *REACTOME_AMINO_ACID_TRANSPORT_ACROSS_THE_PLASMA_MEMBRANE*. This is in line with the known link between amino acid transport and AUT or SCZ—amino acids are essential in cognitive functioning and brain development^49^. For example, increased transport capacity of alanine across the cell membrane and decreased affinity for transport sites of tyrosine may be associated with the development of AUT in children, demonstrating the disturbances in transport mechanisms for amino acids at the membrane level may influence the transport of amino acids across the blood brain barrier^49^. The solute carrier transporter 7a5 (*SLC7A5*), a large neutral amino acid transporter localized at the blood brain barrier, is important in maintaining normal levels of brain branched chain amino acids; the abnormalities in branched-chain amino acid catabolic pathway may cause AUT^50^. The aberrant amino acid transport activities, such as the aberrant tyrosine transport across the cell membrane^51^ and excitatory amino acid transport^52^, were also found in patients with SCZ.

In these five shared gene sets, genes *CLN8, KCNJ10, SLC1A1, SLC1A2, SLC25A12, SLC38A10, SLC6A1, SLC6A8, SLC7A3, SLC7A5*, and *SLC7A7* are overlapped with SFARI AUT-associated genes. Nine of them belong to *SLC* family, of which *SLC1A2* and *SLC6A1* belong to category S (syndromic), and *SLC6A1* is a strong candidate (category 2) for AUT. Among these genes, *SLC1A1* and *SLC1A2* are also associated with SCZ. In particular, gene *SLC1A2* have been linked to SCZ by studies of convergent functional genomics, linkage and association, and differential expression analysis according to the explanation in SZDB^37^. There are 15 more genes associated with SCZ including *ARL6IP5, OCA2, SLC16A12, SLC1A3, SLC1A4, SLC32A1, SLC38A5, SLC38A7, SLC3A1, SLC43A2, SLC6A11, SLC6A17, SLC7A1, SLC7A4*, and *SLC7A6*. Thirteen of these gene belong to *SLC* family, of which *SLC1A3, SLC32A1* and *SLC38A7* are strong candidates for SCZ. Most disease-associated genes in the gene sets belong to *SLC* family, implying the important role of *SLC* in AUT and SCZ.

#### Shared gene sets involved in synapse and neurotransmitter release

Identified gene sets shared by AUT and SCZ also include *REACTOME_NEUROTRANSMITTER_RELEASE_CYCLE*, *GO_SYNAPTIC_VESICLE_RECYCLING*, *GO_SYNAPTIC_VESICLE_ ENDOCYTOSIS*, *GO_GLUTAMATE_SECRETION*, *GO_NEGATIVE_REGULATION_OF_ CATECHOLAMINE_SECRETION*, and *GO_INHIBITORY_SYNAPSE*. The function of brains is dependent on neurotransmission and its alteration is linked to neuropsychiatric disorders. Neurotransmitter release requires the rapid recycling of synaptic vesicles by endocytosis. Evidence has shown synaptic vesicle recycling and endocytosis are related to AUT and SCZ^53–56^. In a study^57^, the identified pathways related to SCZ involved neuronal systems and one hub centered around the neurotransmitter release cycle including the release cycles for dopamine, serotonin, norepinephrine and glutamate neurotransmitters. Glutamate is the main excitatory neurotransmitter of brains and may be a key neurotransmitter involved in neuropsychiatric disorders^58^. Glutamatergic dysfunction is a possible mechanism of AUT^59,60^ and SCZ^61,62^. Catecholamines are monoamine neurotransmitters, including epinephrine, norepinephrine and dopamine. Studies in human neural stem cell models indicated that the dysregulation in catecholamine secretion may contribute to the pathogenesis of AUT^63,64^. It has also been demonstrated that SCZ cases show increased secreted catecholamine and SCZ neuronal cultures show a higher percentage of tyrosine hydroxylase-positive neurons, the first enzymatic step for catecholamine biosynthesis^65^. There also have been studies demonstrating the presence of excitatory and inhibitory imbalance in AUT and SCZ^66,67^, and SCZ-associated cortical inhibitory neurons^68^, thus *GO_INHIBITORY_SYNAPSE* is also likely a related gene set.

In these six shared gene sets, 26 genes are associated with AUT, of which *ALDH5A1, NF1, NTRK2, SLC1A2, SLC6A1* and *STXBP1* belong to category S (syndromic), *NLGN3, RIMS1* and *SLC6A1* belong to category 2 (strong candidate), *CNR1*, *OPHN1* and *STXBP1* belong to category 3 (suggestive evidence) in SFARI. There are 26 genes associated with SCZ, of which *DRD2, CNR1* and *SLC1A2* are the top three strongest candidates, and genes *GABBR1*, *GAD1, IGSF9B, MAOA, SLC1A3, SLC32A1* and *SYN2* have been also linked to SCZ by two kinds of studies in SZDB. Thirteen genes are associated with both AUT and SCZ, including *CNR1, DRD2, GAD1, MAOA, NTRK2, OPHN1, PPFIA1, RIMS1, SLC1A1, SLC1A2, SNAP25, STX1A* and *SYN2*.

#### Shared gene sets involved in oxidative stress and nitric oxide synthase biosynthesis

Two gene sets involved in oxidative stress induced apoptosis were identified to be associated with AUT and SCZ, including *GO_REGULATION_OF_OXIDATIVE_STRESS_INDUCED_INTRINSIC_APOPTOTIC_SIGNALING_PATHWAY* and *GO_NEGATIVE_REGULATION_OF_OXIDATIVE_STRESS_INDUCED_INTRINSIC_APOPTOTIC_SIGNALING_PATHWAY*. The intrinsic apoptotic pathway can be activated following oxidative and peroxidative damage driven by excessive levels of reactive oxygen species (ROS) and reactive nitrogen species (RNS)^69^. When the levels of ROS exceed the antioxidant capacities of a cell, oxidative stress occurs which often leads to the death of a cell^70^. There have been studies showing that oxidative stress is higher in AUT^70^ and SCZ^71^. It also has been shown that oxidative stress can affect the apoptosis of neurons as a mediator in neuropsychiatric disorders, such as SCZ^72^.

Gene sets, associated with nitric oxide synthase biosynthesis, *GO_POSITIVE_REGULATION_OF_ NITRIC_OXIDE_SYNTHASE_BIOSYNTHETIC_PROCESS* and *GO_REGULATION_OF_NITRIC_ OXIDE_SYNTHASE_BIOSYNTHETIC_PROCESS*, are also related to AUT and SCZ. There have been some clues showing the link between nitric oxide synthase biosynthesis and neuropsychiatric disorders. The unbalance between antioxidant capacity and oxidative stress leads to an excess of RNS, such as nitric oxide (NO)^73^. NO, produced by NO synthase (NOS), modulates short-term and long-term synaptic plasticity and is essential in the regulation of many physiological processes such as neurotransmitter release, neuronal excitability, long-term potentiation and neurovascular coupling^74^. NO affects the function of ROS in the local cellular environment, in which biological antioxidants are present. NO shows both neuroprotective and neurotoxic effects. As the disease progresses, NO can depend on the adaptive functions of the antioxidant capacity and oxidative stress-related ROS/RNS^73^. Given the roles of NO, its alteration may lead to the neurodevelopmental changes associated with neuropsychiatric diseases. Indeed, several studies have indicated that NO or NOS is involved in the pathogenesis of many neuropsychiatric disorders including AUT^74–77^ and SCZ^71^.

In these four gene sets, there are two genes associated with AUT, including *GPX1* (glutathione peroxidase 1) and *SOD1* (superoxide dismutase 1), both of which belong to category 4 (minimal evidence). There are 12 genes associated with SCZ, of which *BAG5* and *CPEB1* have been linked to SCZ by two kinds of studies in SZDB. Checking the function of these genes, we found some of them are indeed involved in oxidative stress. The protein encoded by gene *GPX1* catalyzes the reduction of organic hydroperoxides and hydrogen peroxide by glutathione, protecting cells against oxidative damage. The protein encoded by gene *SOD1* is an isozyme responsible for destroying free superoxide radicals in the body, converting naturally generating but harmful superoxide radicals to molecular oxygen and hydrogen peroxide. The protein encoded by gene *BAG5* belongs to *BAG1*-related protein family, and *BAG1* is an anti-apoptotic protein interacting with a variety of proteins involved in cell apoptosis and growth.

#### Shared gene sets involved in immune response

Gene sets *GO_CELL_ACTIVATION_INVOLVED_IN_IMMUNE_RESPONSE* and *GO_LYMPHOCYTE_ACTIVATION_INVOLVED_IN_IMMUNE_RESPONSE* were identified associated with AUT and BPD. Abnormal immunological phenomena have been noted in AUT, involving cytokines, immunoglobulins, inflammation and cellular activation. The increased pro-inflammatory cytokines in brains and the activation of resident immune cells known as microglia in AUT individuals, may interfering with the development and function of normal brains, potentially lead to AUT^78^. In a study^79^, the finding suggested immune activation, including activation of T-lymphocyte subsets, may be essential in modulating and potentially improving behaviors in some AUT patients. It was also found that AUT individuals often have alterations in immune cells, immunoglobulins and autoantibodies. For BPD, the immunological dysfunction was also described, including distinct immune cell profile, release of/altered cytokines by stimulated mononuclear cells, elevated levels of circulating immune markers, inflammatory changes in the central nervous system^80^ and the expansion of activated T cells^81^.

These two shared gene sets contain 10 SFARI AUT candidate genes, and four genes associated with BPD. The 10 genes are *ADA, CX3CR1, FOXP1*, *IFNG*, *IL6, KIT*, *LAT*, *PIK3CG, RORA* and *TSC1. TSC1* belong to category S, and *FOXP1* is also a strong candidate for AUT. There are two genes associated with both AUT and BPD, which are *IFNG* and *RORA*. Both belong to category 5 (hypothesized but untested) in SFARI; nevertheless, the link between these genes and AUT, especially for *RORA*, is well supported^82–84^. Among the four genes associated with BPD, *RORA* and *IFNG* also appear as the strongest candidates for BPD^85–87^.

#### Shared gene sets involved in protein folding

Two gene sets, *GO_DE_NOVO_POSTTRANSLATIONAL_PROTEIN_FOLDING* and *GO_DE_NOVO_PROTEIN_FOLDING*, were identified to be associated with AUT, SCZ and BPD. There have been some clues showing the link between protein folding and neuropsychiatric disorders. Neuroligins (*NLs*), postsynaptic cell-adhesion molecules, are essential for the normal function of synapse. Mutations in neuroligin-4 (*NL4*) (gene symbol: *NLGN4*) have been associated with AUT, for instance R87W substitution (a single amino-acid substitution in *NL4*)^88^. R87W substitution, a loss-of-function mutation, impairs the normal folding of *NL4*, completely traps *NL4* in the endoplasmic reticulum and blocks *NL4* transport to the cell surface. As a result, the synapse formation activity of *NL4* will be inactivated and the functional effect of *NL4* on synapse strength will be blocked^88^.

In the cellular environment, molecular chaperones are required to ensure the correct folding of many other proteins^89^. Heat shock protein (*HSP*) is a kind of molecular chaperone targeting misfolded proteins that accumulate in response to cellular stress, facilitating protein refolding and targeting damaged proteins for degradation in proteasomes^90^. *HSPs* play an essential role in the development of neuropsychiatric disorder, such as *HSP70* (heat shock protein-70). *HSP70* participates in many cellular processes including protein folding, transport across membranes, prevention of protein aggregation and degradation. Genetic variations of *HSP70* have been associated with the presence of SCZ^91^. Looking at the genes contained in these two gene sets, we found seven genes are associated with SCZ, including *CHCHD4, DNAJB1, HSPA8, HSPD1, HSPE1, HSPH1, TOR1A*. Over half of these genes belong to *HSP* family, implying the important role of *HSP* in SCZ.

#### Shared gene sets involved in miscellaneous functions

Gene set *PID_LPA4_PATHWAY* was identified associated with all three diseases. Lysophospholipids (*LPs*) are an important family of lipid signaling molecules, and lysophosphatidic acid (*LPA*) is a major member of this family within the nervous system. *LPA* effects are now known to act through cognate, cell-surface G protein-coupled receptors termed *LPA* receptors (*LPARs*). There are currently six *LPARs*: protein names *LPA*_*1-6*_, gene names *LPAR*_*1-6*_. Large evidence has been shown that *LPA*_*1*_ signaling is essential in normal cognition. For instance, *LPAR*_*1*_ null mice display a variety of negative behavioral signs and cognitive deficits including the traits commonly seen in AUT and SCZ patients. *LPA* is also known to reduce glutamate uptake involving an *LPA*_*1*_-independent mechanism while glutamatergic signaling alterations are implicated in AUT, SCZ and other related neuropsychiatric disorders^92^. *LPA*_*1*_ has been particularly associated with neuropsychiatric disorders, while other *LPA* receptor subtypes may also have disease relevance^93^, which may provide new insights into complex neuropsychiatric disorders. In the gene set, genes *ADCY3* and *ADCY5* are associated with AUT; *ADCY8* and *RPS6KA5* are associated with SCZ; *ADCY2*, *ADCY8*, *ADCY9*, and *CREB1* are associated with BPD.

Gene set *REACTOME_GLYCOLYSIS* was identified associated with AUT and SCZ. The majority of glucose metabolites are significantly disturbed in SCZ patients, suggesting the disturbance of glucose metabolism may be implicated in SCZ^94,95^. Additionally, the findings of a recent study in 2018 have also suggested an elevation of glycolysis through the phenomenon of aerobic glycolysis in AUT, while the dysregulation of aerobic glycolysis had been proposed as a candidate cause of AUT^96^. In the gene set, genes *PPP2R1B* and *PPP2R5D* belong to category 4 (minimal evidence) in SFARI, and *PPP2R5D* also belongs to category S. Genes *ALDOA, ALDOB, PFKFB1, PGK1* and *PPP2CA* are linked to SCZ.

Gene set *GO_CAMP_BIOSYNTHETIC_PROCESS* was associated with AUT and BPD. *cAMP* (cyclic adenosine monophosphate, or cyclic *AMP*) is a second messenger, which is important in many biological processes. There have been studies showing the link between *cAMP* and AUT, such as the study showing reduced *cAMP* induction may be a cause of fragile X and AUT^97^, and the study pointing out the role of *cAMP* pathology in AUT^98^. Furthermore, a genetic association study of *cAMP* signaling genes with BPD found several statistically significant single-SNP associations and SNP-SNP associations with BPD, suggesting that variants in several *cAMP* signaling pathway genes increase the risk of BPD^99^. In the gene set, genes *ADCY3* belongs to category 3 (suggestive evidence), and *ADCY5* and *ADORA2A* belong to category 4 (minimal evidence) in SFARI. Genes *ADCY2, ADCY8, ADCY9*, and *ADM* are associated with BPD.

Gene set *ST_PAC1_RECEPTOR_PATHWAY* is associated with AUT and SCZ*. PAC1* is a receptor of *PACAP* (pituitary adenylate cyclase-activating polypeptide, a neuropeptide with neurotransmission modulating activity). *PACAP* and its receptor *PAC1* are important for the development and function of brains, psychiatric conditions and stress response^100^. Studies raised the potential relation between *PACAP* signaling dysfunctions and neuropsychiatric disorders characterized by social reciprocity impairments such as AUT^101^. *PAC1* gene contains many putative splicing factor recognition sites which might be activated at different stages of neuronal activation. *PAC1* signaling controls many cellular and physiological responses, such as proliferation, differentiation, cell cycle regulation, neurotransmitter, and hormone release and adaptation to stressful challenges^102^. The regulation of *PAC1* splicing and its outcomes might be relevant to the etiology of some neurological and psychiatric disorders^102^. A genetic study^103^ showed that the variants of the genes encoding *PACAP* and *PAC1* receptor are associated with SCZ. The transcriptome sequencing of the cortex of SCZ patients revealed significant differences in the alternative splicing of *PAC1* receptor^104^. The evidences above show the link of *PAC1* receptor pathway to neuropsychiatric disorders.

### Results obtained using consensus co-expression analysis

**Fig. 3:**
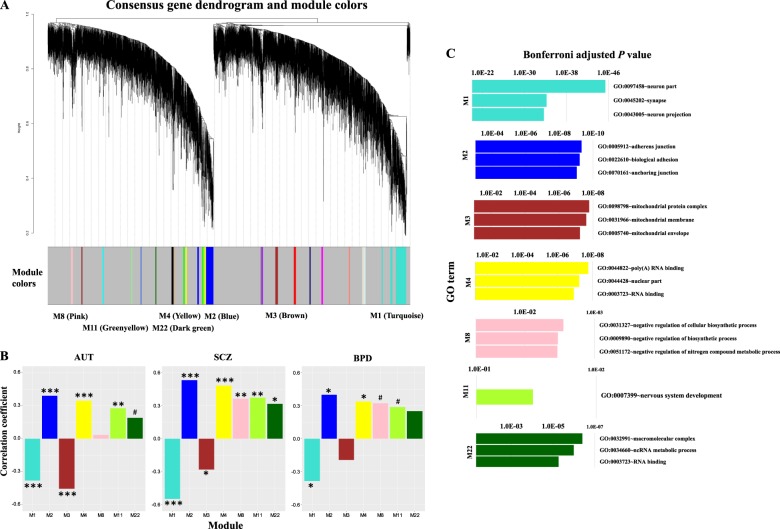
**a** The clustering tree (dendrogram) of genes and the identified consensus modules. The mentioned modules (M1, M2, M3, M4, M8, M11 and M22) in the main text are marked. For these modules, **b** shows the correlation between module eigengenes and disease status. FDR-corrected *P* < 0.001(***); <0.01 (**); <0.05 (*); <0.1 (#). **c** The top three significant GO terms enriched in responding modules are shown with Bonferroni adjusted *P*-values

In addition to aberrant gene expression analysis, we performed gene co-expression analysis using WGCNA^44^. Specifically, after obtaining final data matrix containing the expression level of 8,485 protein-coding genes in 186 samples (47 AUT, 31 SCZ, 25 BPD and 83 controls), we firstly split the data into three sub-datasets, denoted as datasets 1, 2, and 3, each of which contains respective cases and controls (Fig. 1). Dataset 1 contains the gene expression level of 47 AUT and 57 controls from data I; dataset 2 contains 31 SCZ and 26 controls from data II; dataset 3 contains 25 BPD and 26 controls from data II. Note that, SCZ and BPD cases are originally from data II, so the respective controls are also from data II. Next, we applied WGCNA to find consensus modules between datasets 1, 2 and 3, and 22 modules were identified (Materials and Methods, Fig. 3a). As the 26 controls included in datasets 2 and 3 are shared, we used a strategy similar to that in the study of Ellis et al.^32^, where these controls were split randomly into two halves. Specifically, we divided the 26 controls randomly into two halves, and one half was assigned to a new dataset 2 along with all SCZ cases, and another half was assigned to a new dataset 3 along with all BPD cases. Then we reconstructed networks to find consensus modules with the same parameters between dataset 1, new datasets 2 and 3. The procedure above was repeated for 100 times. To demonstrate the robustness of the consensus modules built in full data with datasets 2 and 3 containing 26 controls respectively, we used a similar method in a previous study^46^ showing the clustering tree (dendrogram) of genes together with consensus modules built from the full data and the 100 ones built from the resampled data with new datasets 2 and 3 containing 13 controls respectively (Supplementary Figure 1**)**. Most of the 22 identified consensus modules built in full data are robust and can be identified in most or all resampled data sets, so we used these 22 consensus modules as the final module assignments.

We associated each consensus module with traits (age, gender and disease status) (Materials and Methods) and found 17 modules are shared by more than one diseases (FDR-corrected *P*-value < 0.1) (Supplementary Table 2). We used the database for annotation, visualization and integrated discovery (DAVID)^47,48^ to perform gene ontology analysis and seven modules left were enriched in GO categories. Modules M1, M2, M4 and M11 are associated with all disorders; M3 and M22 are shared by AUT and SCZ; M8 is shared by SCZ and BPD (Fig. 3b). The top three significant GO terms enriched in these modules include neuron part, synapse, neuron projection, adherens junction, biological adhesion, anchoring junction, poly(A) RNA binding, RNA binding, nuclear part, nervous system development, mitochondrion, macromolecular complex, ncRNA metabolic process, and negative regulation of biosynthetic and nitrogen compound metabolic processes (Fig. 3c).

Comparing aberrant gene expression analysis with WGCNA, we found functions of gene sets identified by these two methods are similar and largely overlapped (Supplementary Table 3). There are 301 unique genes included in the 30 shared aberrantly expressed gene sets and 736 genes included in the seven shared WGCNA gene modules. Among them, 52 genes were overlapped and their functions include many of those related to synapse, neuron, cell projection, localization, cell-cell signaling and so on (Supplementary Table 3). Thus, aberrant gene expression analysis and WGCNA produce consistent results and are complementary to each other to guarantee the identification of comprehensive gene sets associated with neuropsychiatric disorders.

## Discussion

Between AUT, SCZ and BPD, similar clinic symptoms and shared genetic etiology have been reported^3–7^. With the availability of human brain transcriptome data from postmortem donors affected with different neuropsychiatric disorders, gene expression anlaysis has been widely used to identify shared genetic component underlying different disorders; nevertheless, depending on the analytic methods adopted, the results of analysis may vary, each capturing different features of underlying mechanisms of dysregulation. For example, Ellis et al. analyzed the transcriptomic RNA-sequencing data of cortex samples of AUT, SCZ, BPD and controls from two previous study^24,31^ using single gene-based differential expression analysis. They found the transcriptomes of AUT and SCZ are correlated, and altered neurotransmission and synapse regulation are shared between these two disorders^32^. Their analysis method focused on obtaining single differentially expressed genes for each disease and then finding the shared ones across disorders. Different from that, here we used multivariate analysis methods to re-analyze the gene expression data for identifying shared dysregulated gene sets across AUT, SCZ and BPD.

The most common gene expression analysis method for identifying shared genes across disorders is based on differential gene expression analysis. Differential gene expression analysis is used to detect the genes with significant difference in gene expression means between diseased and control samples and then the shared differentially expressed genes across diseases are identified. Except from the difference of gene expression means between groups, the difference of gene expression variability also need to be captured. In addition, the major assumption underlying differential expression analysis is: diseased cases have the same or similar gene expression change phenotypes, which makes them as a separate group have significantly higher or lower gene expression than the controls. This assumption contradicts the fact that neuropsychiatric disorders have substantial genetic and phenotypic heterogeneity. Therefore, we need to capture the gene expression variability in diseased samples affected by complex neuropsychiatric disorders. For this, we applied aberrant gene expression analysis method^39^, quantitatively measuring the departure of multigene expression dispersion between groups, to identify shared dysregulated gene sets across AUT, SCZ and BPD. The identified shared gene sets include the ones associated with amino acid transport activity, neurotransmitter release, synaptic vesicle, excitatory and inhibitory synapse, oxidative stress, nitric oxide synthase biosynthesis, immune response, protein folding, lysophosphatidic acid-mediated signaling and glycolysis. Our method is not dependent on the prior knowledge about gene function or mutations in genes. Thus, it can be used for discovering and identifying genes or gene sets previously unknown to be involved in the progression of AUT, SCZ and BPD. Aberrant gene expression analysis is effective in discovering and revealing shared dysregulated gene sets across disorders, contributing to the study of gene expression overlap between AUT, SCZ and BPD.

In addition to aberrant gene expression analysis, we also applied weighted gene co-expression network analysis to identify shared gene modules across disorders. The functions of several identified modules have been associated with neuropsychiatric disorders. For instance, the modules shared by AUT, SCZ and BPD are enriched for GO terms related to neuron, synapse, adherens junction, RNA binding and nervous system development. For adherens junction, there has been literature documenting the links with AUT, SCZ and BPD^105–108^. Adherens junctions are cadherin-based intercellular adhesions^109^ and the cadherin genes implicated in psychiatric disorders were overrepresented in cell-cell adhesion and adherens junction organization^108^. There also have been many studies documenting the contribution of dozens of RNA binding proteins to neurodegenerative and neurodevelopmental disorders such as AUT and SCZ^110^. For example, the loss of function of *FMRP*, a polyribosome-associated neuronal RNA-binding protein, causes Fragile X syndrome and autistic features. *FMRP* interacts with the coding region of transcripts which encode pre- and postsynaptic proteins and transcripts implicated in AUT. *FMRP* target genes significantly overlap with AUT candidate genes in SFARI^111^. Recently, a paper has studied the link between poly(A) binding protein nuclear I and synaptic plasticity^112^, implying the relation between poly(A) RNA binding and neuropsychiatric disorders. Another WGCNA gene module, shared by AUT and SCZ, is enriched for GO terms related to mitochondrion. Large literature reported the mitochondrial dysfunction in AUT and SCZ, including evidences of decreased activity of mitochondrial respiratory chain complexes, the presence of biomarkers of oxidative stress and mitochondrial dysfunction and an indication of mtDNA mutations^113–116^. Comparing aberrant gene expression analysis and WGCNA, we found the functions of gene sets identified by these two kinds of methods are similar and overlapped. These two kinds of analysis methods complement to each other and promote to identify more comprehensive gene sets associated with neuropsychiatric disorders.

To identify genetic overlap between AUT, SCZ, and BPD, we applied multigene aberrant expression analysis, along with consensus co-expression network analysis, to identify shared dysregulated gene sets in cortical brains of individuals affected with different diseases. Our findings provide new insights into the common molecular mechanisms underlying the pathogenesis and progression of AUT, SCZ and BPD, contributing to the study of etiological overlap between these neuropsychiatric disorders. We show that the aberrant gene expression analysis reveals the variability in gene expression among diseased samples. This method, complementing with the network-based method, is effective in detecting dysregulated gene sets shared across neuropsychiatric disorders, and is applicable in genetic overlap analysis for other complex diseases.

## Supplementary information

Supplementary Figure 1

Supplementary Table 1

Supplementary Table 2

Supplementary Table 3

Supplementary Data 1

## Data Availability

Computer codes used throughout for data pre-processing and data analyses are available from corresponding author.
